# Islet cell hyperexpression of HLA class I antigens: a defining feature in type 1 diabetes

**DOI:** 10.1007/s00125-016-4067-4

**Published:** 2016-08-09

**Authors:** Sarah J. Richardson, Teresa Rodriguez-Calvo, Ivan C. Gerling, Clayton E. Mathews, John S. Kaddis, Mark A. Russell, Marie Zeissler, Pia Leete, Lars Krogvold, Knut Dahl-Jørgensen, Matthias von Herrath, Alberto Pugliese, Mark A. Atkinson, Noel G. Morgan

**Affiliations:** 1 Islet Biology Exeter (IBEx), Institute of Biomedical and Clinical Sciences, University of Exeter Medical School, RILD Building (Level 4), Barrack Road, Exeter, EX2 5DW UK; 2 La Jolla Institute for Allergy and Immunology, San Diego, CA USA; 3 Department of Medicine, University of Tennessee, Memphis, TN USA; 4 Department of Pathology, University of Florida, Gainesville, FL USA; 5 Department of Information Sciences, City of Hope, Duarte, CA USA; 6 Paediatric Department, Oslo University Hospital, Oslo, Norway; 7 Faculty of Medicine, University of Oslo, Oslo, Norway; 8 Diabetes Research Institute, Department of Medicine, Division of Diabetes, Endocrinology and Metabolism, University of Miami Miller School of Medicine, Miami, FL USA; 9 Department of Microbiology and Immunology, University of Miami Miller School of Medicine, Miami, FL USA

**Keywords:** DiViD, HLA class I, HLA-F, Islet cell, nPOD, Pancreas, STAT1, Type 1 diabetes

## Abstract

**Aims/hypothesis:**

Human pancreatic beta cells may be complicit in their own demise in type 1 diabetes, but how this occurs remains unclear. One potentially contributing factor is hyperexpression of HLA class I antigens. This was first described approximately 30 years ago, but has never been fully characterised and was recently challenged as artefactual. Therefore, we investigated HLA class I expression at the protein and RNA levels in pancreases from three cohorts of patients with type 1 diabetes. The principal aims were to consider whether HLA class I hyperexpression is artefactual and, if not, to determine the factors driving it.

**Methods:**

Pancreas samples from type 1 diabetes patients with residual insulin-containing islets (*n* = 26) from the Network for Pancreatic Organ donors with Diabetes (nPOD), Diabetes Virus Detection study (DiViD) and UK recent-onset type 1 diabetes collections were immunostained for HLA class I isoforms, signal transducer and activator of transcription 1 (STAT1), NLR family CARD domain containing 5 (NLRC5) and islet hormones. RNA was extracted from islets isolated by laser-capture microdissection from nPOD and DiViD samples and analysed using gene-expression arrays.

**Results:**

Hyperexpression of HLA class I was observed in the insulin-containing islets of type 1 diabetes patients from all three tissue collections, and was confirmed at both the RNA and protein levels. The expression of β2-microglobulin (a second component required for the generation of functional HLA class I complexes) was also elevated. Both ‘classical’ HLA class I isoforms (i.e. HLA-ABC) as well as a ‘non-classical’ HLA molecule, HLA-F, were hyperexpressed in insulin-containing islets. This hyperexpression did not correlate with detectable upregulation of the transcriptional regulator NLRC5. However, it was strongly associated with increased STAT1 expression in all three cohorts. Islet hyperexpression of HLA class I molecules occurred in the insulin-containing islets of patients with recent-onset type 1 diabetes and was also detectable in many patients with disease duration of up to 11 years, declining thereafter.

**Conclusions/interpretation:**

Islet cell HLA class I hyperexpression is not an artefact, but is a hallmark in the immunopathogenesis of type 1 diabetes. The response is closely associated with elevated expression of STAT1 and, together, these occur uniquely in patients with type 1 diabetes, thereby contributing to their selective susceptibility to autoimmune-mediated destruction.

**Electronic supplementary material:**

The online version of this article (doi:10.1007/s00125-016-4067-4) contains peer-reviewed but unedited supplementary material, which is available to authorised users.

## Introduction

The incidence of type 1 diabetes is increasing rapidly worldwide [1–3], probably because of changes in the environment that ultimately impact the development, functional activity and longevity of pancreatic beta cells.

Against this background, the cellular and molecular events associated with the initiation and progression of type 1 diabetes remain poorly understood, largely because the disease process cannot be studied non-invasively in the pancreases of living individuals. Hence, deductions regarding pathogenic processes are made from analyses of tissue recovered either after death or by pancreas biopsy in living individuals [4–7]. Collectively, such approaches have been applied in few cases worldwide, reflecting the paucity of accessible samples in which the tissue architecture has been preserved and the destructive process is still present and amenable to study [8, 9].

Despite these limitations, a consensus model has emerged in which type 1 diabetes is envisaged to result from the selective destruction of beta cells by immune cells infiltrating the islets of Langerhans [1–3, 8, 9]. In this scenario, CD8^+^ T cells are considered to be the major effectors of beta cell death, and are directed to the pancreatic islets to participate in the autoimmune assault against beta cells [10–13]. It is also likely, however, that beta cells are complicit in these events by aberrantly processing and presenting cellular antigens, thereby becoming visible to autoreactive CD8^+^ T cells [14]. This could be achieved in several ways, including via upregulated expression of the MHC (i.e. HLA class I [HLA-I]) molecules [15–17].

Upregulation of HLA-I expression (often cited as ‘hyperexpression’) in pancreatic islets has been studied in relatively few type 1 diabetes patients, and no previous attempts have been made to verify the phenomenon across multiple cohorts. Moreover, the concept has primarily been examined at the protein level using immunocytochemical approaches and has rarely been corroborated with gene-expression data to verify that the two are concordant. Indeed, in one recent study, it was argued that such concordance may not exist [18].

Therefore, in the present work, we have taken advantage of a unique collaborative strength achieved by combining three of the world’s most significant collections of pancreas samples from individuals with type 1 diabetes: an archival collection of postmortem samples from the UK [4]; the Network for Pancreatic Organ donors with Diabetes (nPOD) collection of organ donor pancreases (USA) [19, 20]; and pancreatic biopsy material from living individuals participating in the Norwegian Diabetes Virus Detection study (DiViD) [7]. In this study, we interrogated these tissues to provide definitive evidence as to whether islet cell hyperexpression of HLA-I antigens is an early and defining feature of human type 1 diabetes. Collectively, these new data allow us to report the most comprehensive examination of HLA-I expression ever undertaken involving the human pancreas in type 1 diabetes.

## Methods

### Tissue

Formalin-fixed, paraffin-embedded (FFPE) pancreas sections were available from three cohorts: the nPOD and DiViD collections, and an archival collection from the UK (electronic supplementary material [ESM] Tables 1, 2). Frozen tissue was also available from nPOD and DiViD. Analyses were performed with 17 control and 26 type 1 diabetic individuals for whom FFPE and frozen tissue were available. All samples were studied with appropriate ethical approval and, in the case of the DiViD study, participants provided written informed consent.

### Immunohistochemistry and immunofluorescence

Immunohistochemistry was performed using a standard immunoperoxidase approach, as previously described [21]. To examine multiple antigens within the same FFPE section, samples were probed in a sequential manner with up to three different antibodies (ESM Tables 3, 4). The mean fluorescence intensity (MFI) of stained antigens was measured using ImageJ Version 1.50b Java 1.8.0_77; https://imagej.nih.gov/ij/download.html. Some slides were processed with isotype control antisera to confirm the specificity of labelling (ESM Fig. 1). Frozen sections were stained using a standard immunofluorescence approach [22].

### Islet microdissection and RNA collection

Optimal cutting temperature (OCT) slides of pancreatic tissue were used for laser-capture microscopy. This was conducted on an Arcturus Pixcell II laser capture microdissection system (Arcturus Bioscience, Mountain View, CA, USA). Islets were recognised by their natural autofluorescence [23]. All islets visible in two to five sections from each sample were pooled, and RNA was extracted using the Arcturus PicoPure RNA Isolation Kit (Applied Biosystems, Grand Island, NY, USA). RNA quantity and quality were determined using a Bioanalyzer 2100 (Agilent Technologies, Santa Clara, CA, USA). RNA samples were subjected to gene-expression analysis using Affymetrix expression arrays (Thermo Fisher Scientific, Santa Clara, CA, USA), as previously described [24].

### Affymetrix array analysis

Using the Affymetrix Human Gene 2.0 ST array, CEL files were generated from both control and type 1 diabetic donors, as previously described [24]. Raw signal-intensity values from Affymetrix spike-in controls demonstrated that array hybridisation had been successful (i.e. bioB<bioC<bioD<Cre). Data quality was verified by measuring the positive vs negative area under the curve. Raw signal-intensity values from all arrays were robust multichip average background corrected, quantile normalised, median polish summarised and log_2_ transformed [25–27]. NetAffx-determined probe-set annotations for HLA genes (Affymetrix) were re-mapped according to RefSeq, release 73 (15 November 2015; see ftp://ftp.ncbi.nlm.nih.gov/refseq/release/release-catalog/archive/). For each HLA gene, where multiple mappings were possible (i.e. *HLA-A*, *-B*, *-C* and *-F*), probe sets were annotated according to eight major haplotypes incorporated into the human genome assembly, as previously described [28]. Because probe sets shared mappings, it was not possible to identify HLA subtypes uniquely using this gene chip; rather, transcript clusters were used to examine changes in global gene expression. Processing was carried out using the Partek Genomics Suite, version 6.5 (Partek, St Louis, MO, USA). The resulting normalised expression data for specific genes of interest were then subjected to analysis as described below.

### Statistical analysis

Individual comparisons of MFI, RNA or protein were performed using either a Satterthwaite corrected two-sample test or paired/unpaired Student’s *t* test and considered significant if *p* < 0.05. In the case of multiple comparisons, statistical significance was indicated at a Bonferroni-corrected nominal α level of 0.025. Correlations were evaluated and considered strong if *p* < 0.05 and the Spearman’s rank correlation coefficient (*r*) was >0.80. All reported *p* values are two-tailed and unadjusted. Statistical analyses were performed using SAS version 9.4 (SAS Institute, Cary, NC, USA).

## Results

### Lobular hyperexpression of HLA-A, -B and -C (HLA-ABC) in type 1 diabetes

**Fig. 1:**
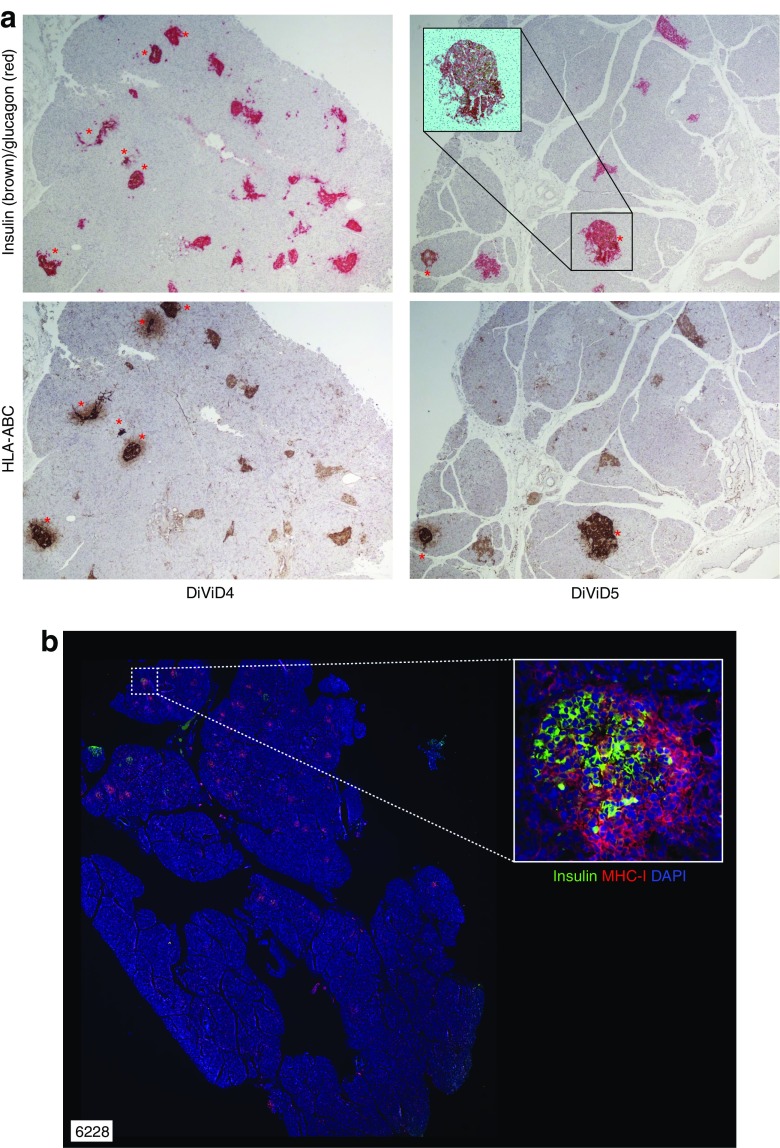
Immunocytochemical analysis of the expression HLA-ABC in pancreas tissue. (**a**) Pancreas sections from two individuals with recent-onset type 1 diabetes from the DiViD cohort showing insulin and HLA-ABC immunostaining on serial sections. ICIs are indicated with red asterisks (magnification ×40 for the whole tissue section and ×100 for the islet). (**b**) Immunofluorescence analysis of HLA-ABC expression in frozen pancreas from a patient with recent-onset type 1 diabetes from the nPOD cohort. Hyperexpression of HLA-ABC (red) was predominantly seen only in ICIs (green; inset) (magnification ×40 for the whole tissue section and ×400 for the islet)

In accord with earlier reports [9, 14, 21, 22], hyperexpression of HLA-ABC was consistently observed in the islets of patients with type 1 diabetes among all cohorts examined (Fig. 1), but not in controls. The pattern was lobular and mainly restricted to insulin-containing islets (ICIs) (Fig. 1), while insulin-deficient islets (IDIs) displayed normal expression. Islet hyperexpression of HLA-ABC was not confined solely to beta cells, but occurred in all islet endocrine cells (Fig. 1, ESM Fig. 1b).

### Classification of donors based on HLA-ABC expression

Since islet hyperexpression of HLA-ABC has been claimed to be artefactual [18], we monitored the levels of HLA-ABC in a subset of nPOD donors in two independent laboratories using pancreas sections preserved by different methods (frozen vs FFPE). Staining for HLA-ABC was performed using either an immunoperoxidase method coupled with a mouse primary antiserum in FFPE tissue (ESM Fig. 2a) or via immunofluorescence in OCT sections (ESM Fig. 2b) from the same donor, using a different primary antiserum. A blinded analysis was conducted with donors classified into three categories: normal, elevated and hyperexpression (i.e. at least one islet with extremely high expression of HLA-ABC affecting all endocrine cells) (ESM Fig. 2). Unblinding of the analysis revealed a 100% concordance rate between laboratories (ESM Fig. 2c).

Further confirmation of the staining specificity in FFPE tissue was obtained by staining serial islet sections with two different HLA-ABC antibodies. In all cases where hyperexpression of HLA-ABC was detected with one antiserum, this was confirmed in the same islet on the serial section with the second antiserum (ESM Fig. 3, ESM Tables 3, 4).

Examination of patients with increasing disease duration revealed that HLA-ABC hyperexpression was not restricted only to patients with recent-onset type 1 diabetes, but was also observed in individuals with longer-term disease (i.e. up to 11 years) when ICIs were retained (ESM Fig. 4). However, the proportion of ICIs hyperexpressing HLA-ABC decreased as the duration of type 1 diabetes increased (*r* = –0.883, *p* < 0.0001; ESM Fig. 4). HLA-I hyperexpression was not found in patients lacking residual ICIs. It was also absent from the ICIs of patients with still longer disease durations (>11 years), even among those who retained insulin immunopositivity (three nPOD patients with a total of 110 ICIs) after this time (ESM Fig. 4).

### β2-Microglobulin (β2M) is elevated in islets in type 1 diabetes

**Fig. 2:**
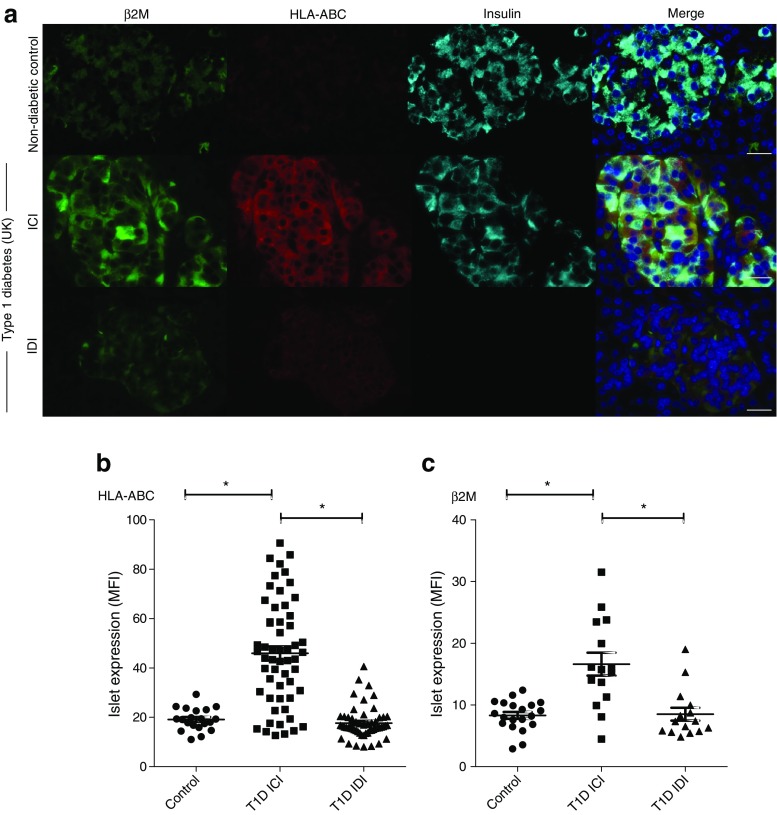
Correlation between HLA-ABC, β2M and insulin expression in controls and individuals with type 1 diabetes. (**a**) Analysis of β2M (green), HLA-ABC (red) and insulin (light blue) in an ICI from a non-diabetic control individual and a type 1 diabetes patient, and an IDI from the same individual. Scale bar, 25 μm. (**b**) The MFI of islet HLA-ABC expression was measured in 5–14 islets among non-diabetic control individuals (*n* = 4) and in the ICI and IDIs of nine individuals with type 1 diabetes (T1D) (five from the DiViD cohort, three from the UK cohort and one from the nPOD cohort). (**c**) The MFI of islet β2M expression was measured in 15 ICIs and 15 IDIs from each of three type 1 diabetes patients (two from the UK cohort and one from the nPOD cohort). This was compared with the expression in 20 islets from each of four non-diabetic control individuals (three from the UK cohort and one from the nPOD cohort). *p < 0.001

Functional HLA-I complexes are heterodimers comprised of an isoform of HLA-I plus β2M. Therefore, the levels of β2M were also assessed and found to be expressed differentially in patients with type 1 diabetes and controls. β2M was present in the islets of individuals without type 1 diabetes (Fig. 2a), but its expression was increased in the ICIs of patients with type 1 diabetes, which also hyperexpressed HLA-ABC. IDIs from the same individuals expressed levels of β2M and HLA-ABC comparable with those seen in non-diabetic controls (Fig. 2b, c).

### Expression of RNA transcripts encoding HLA or *β2M* in laser-captured, microdissected islets

**Fig. 3:**
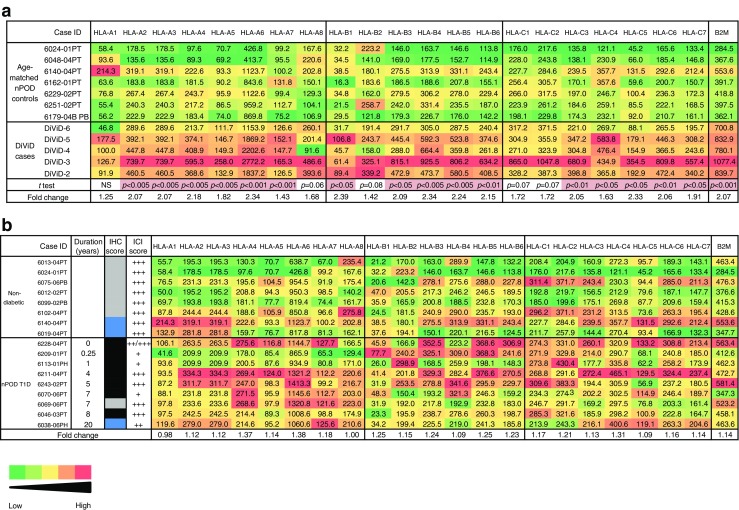
Heat map illustrating the relative expression of *HLA-ABC* and *B2M* genes in control individuals and those with type 1 diabetes (T1D). The expression of each probe set is displayed separately in islets of: (**a**) seven nPOD non-diabetic controls age-matched to five DiViD patients; and (**b**) eight nPOD non-diabetic controls and nine nPOD type 1 diabetic donors. Expression values are shown in arbitrary units and the heat map illustrates relative expression ranging from low (green) to high (red). In (**b**), a comparison with the level of expression scored after immunohistochemical analysis of islets present in nearby pancreatic blocks from the same patients is provided (black, hyperexpression; blue, elevated expression; grey, normal expression), together with an indication of the extent of insulin immunopositivity

Next, the expression of HLA isoforms and *β2M* was examined at the RNA level in laser-captured, microdissected islets. RNA was extracted from pooled islets harvested in a manner that did not differentiate between islets with hyperexpression or normal expression of *HLA-I* or between ICIs and IDIs (Fig. 3). Initially, RNA expression profiles were analysed in islets from the DiViD cohort, since these represent patients with recent-onset disease who retained ICIs [29, 30]. Age-matched control individuals were selected from the nPOD collection. When displayed in a ‘heat map’ format to indicate relative RNA levels using multiple probe sets (Fig. 3a), each of the HLA isoforms (*HLA-ABC*) and *β2M* were shown to be markedly elevated. Quantification yielded mean ± SEM increases of 1.9 ± 0.14-fold, 2.15 ± 0.16-fold, 2.02 ± 0.09-fold for *HLA-A*, *-B* and *-C*, respectively, and 2.07-fold for β2M. Data from the nPOD cohort revealed similar trends (ESM Fig. 5), although the effects were less marked. When analysis of the nPOD population was refined by excluding individuals in whom no ICIs could be found in sections adjacent to the pancreatic blocks used for islet RNA isolation, the trend for increased expression of *HLA-ABC* and *β2M* was more pronounced (Fig. 3b).

### *HLA-F* expression is also elevated in the ICIs of individuals with recent-onset type 1 diabetes

**Fig. 4:**
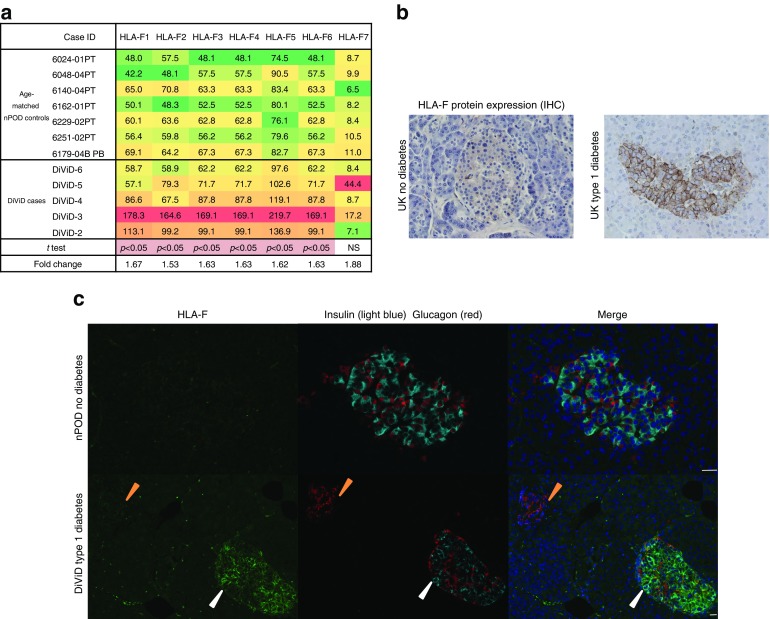
Expression of HLA-F in control individuals and type 1 diabetes patients. (**a**) Heat map illustrating the relative expression of the HLA-F probe sets in seven nPOD non-diabetic controls age-matched to five DiViD patients. (**b**) Representative immunostaining of islets from an individual without diabetes and a patient with type 1 diabetes with anti-HLA-F. (**c**) Immunofluorescence staining of HLA-F (green), insulin (light blue) and glucagon (red) in an ICI (white arrowheads) and an IDI (orange arrowheads) of a DiViD type 1 diabetes patient, and an islet from an nPOD control donor. Scale bar, 25 μm

During analysis of RNA expression in islets from the DiViD patients, it was observed that a non-classical HLA, *HLA-F*, was also upregulated (by 1.71 ± 0.04-fold) when analysed across all probe sets (Fig. 4a). Therefore, expression at the protein level was assessed in FFPE tissue. This revealed that HLA-F is expressed at low levels in the islets of non-diabetic controls, but is upregulated in the ICIs of patients with recent-onset type 1 diabetes (Fig. 4b, ESM Fig. 6a). The elevated expression was not restricted to beta cells, but could also be observed in alpha cells (Fig. 4c). Similar findings were observed in pancreas tissue from the nPOD, DiViD and UK cohorts (ESM Fig. 6b). Surface localisation of HLA-ABC and HLA-F was observed, but HLA-ABC was also seen in the cytosol of ICIs (ESM Fig. 6c).

### NLR family CARD domain containing 5 (NLRC5) expression does not correlate with HLA-ABC hyperexpression

**Fig. 5:**
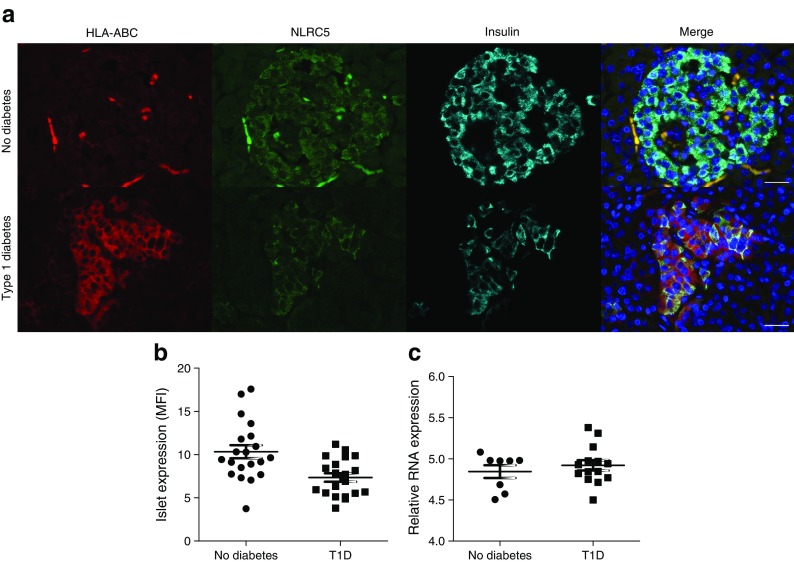
Expression of NLRC5 in the islets of control individuals and those with type 1 diabetes. (**a**) Representative islets from an individual without diabetes and a patient with type 1 diabetes are shown (red, HLA-ABC; green, NLRC5; light blue, insulin; dark blue, DAPI). Scale bar, 25 μm. (**b**) MFI values for NLRC5 protein expression were quantified after immunostaining in five islets per section from four control and four type 1 diabetes (three from the UK, one from nPOD) samples (*p* = 0.0704). (**c**) Expression of *NLRC5* was compared in RNA isolated from islets of individuals with and without type 1 diabetes (*p* = 0.4504)

In order to understand the factors that might drive islet HLA-I hyperexpression in type 1 diabetes, we studied NLRC5, a known transcriptional regulator of HLA-ABC and β2M [31]. NLRC5 was readily detected at the protein level in the cytoplasm of beta cells in healthy control pancreas (Fig. 5a). Expression of NLRC5 was similarly detected in the islets of patients with type 1 diabetes but was not elevated, even in islets with demonstrably elevated HLA-ABC expression (Fig. 5a,b). This was confirmed at the RNA level in laser-captured, microdissected islets (Fig. 5c; *p* = 0.4504).

### Signal transducer and activator of transcription 1 (STAT1) expression correlates positively with HLA-ABC hyperexpression in type 1 diabetes

**Fig. 6:**
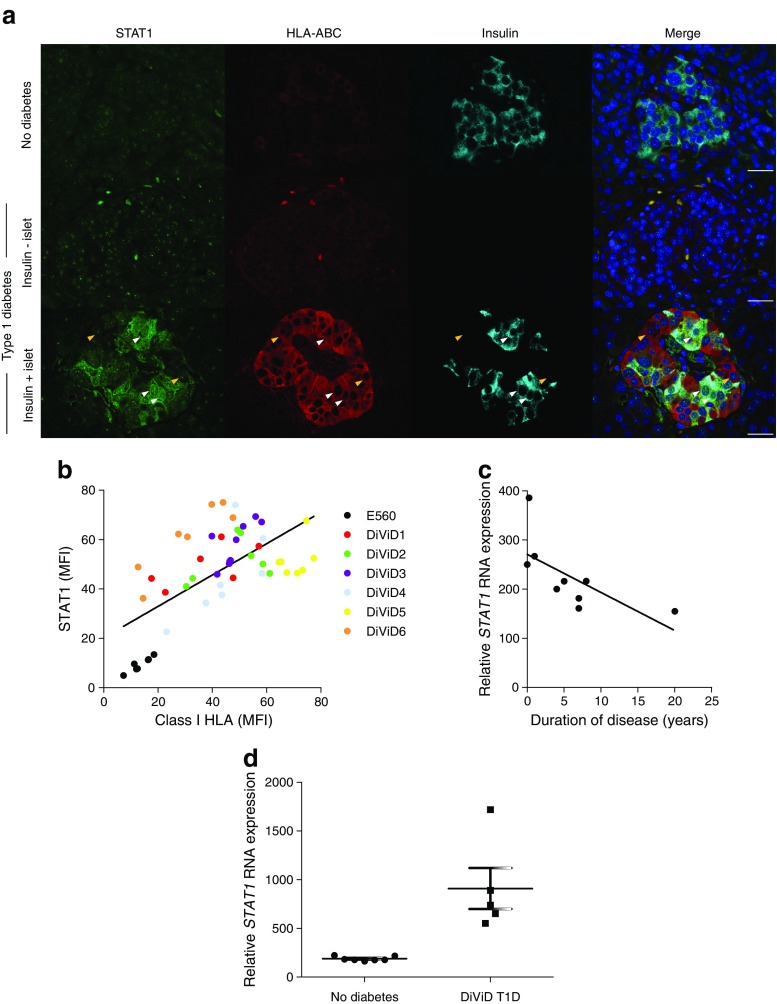
Expression of STAT1 and HLA-ABC in islets from control individuals and those with type 1 diabetes. (**a**) Representative islets from a control individual and from a type 1 diabetes patient were immunostained for STAT1 (green), HLA-ABC (red), insulin (light blue) and DAPI (dark blue). The localisation of STAT1 is shown in beta cells (white arrowheads) and non-beta cells (orange arrowheads). Scale bar, 25 μm. (**b**) MFI values for STAT1 and HLA-ABC expression were quantified and correlated from a minimum of seven ICIs in seven patients with type 1 diabetes (T1D) among the DiViD and UK (E560) cohorts (Spearman’s rank coefficient =0.5454, *p* < 0.0001). (**c**) Correlation between the expression of *STAT1* mRNA and disease duration in nPOD type 1 diabetes patients with residual ICIs (*p* < 0.05). (**d**) Analysis of *STAT1* expression in RNA isolated from islets from DiViD patients and age-matched control donors (nPOD) (*p* = 0.0263)

Given that NLRC5 expression was not found to change in parallel with HLA-ABC or β2M in the islets of patients with type 1 diabetes, a second transcriptional regulator, STAT1, was investigated. This protein was present at low levels in the islets of non-diabetic controls (Fig. 6a), and the pattern of staining was similar in tissues from each of the three patient cohorts examined (UK, nPOD and DiViD). STAT1 expression was also low in the IDIs of type 1 diabetes donors (Fig. 6a). However, STAT1 levels were markedly elevated in ICIs that hyperexpressed HLA-ABC (Fig. 6a). STAT1 expression was highest in beta cells and appeared to be localised within both the cytoplasm and the nucleus (Fig. 6a). The fluorescence intensity for immunolabelling of STAT1 and HLA-ABC was measured across a minimum of seven ICIs in seven different individuals. This revealed a striking positive correlation between STAT1 and HLA-ABC expression (Fig. 6b, overall Spearman’s *r* = 0.5454, *p* < 0.0001).

Analysis of the expression of *STAT1* at the mRNA level in laser-captured islets confirmed the data obtained at the protein level. Moreover, in common with HLA-ABC (ESM Fig. 4), the extent of this increase declined with disease duration (Fig. 6c). Consistent with this, the most pronounced rise was seen in islets harvested by laser-capture microdissection from the six DiViD patients (*p* = 0.0263; Fig. 6d, ESM Fig. 7), who were studied very close to disease onset.

## Discussion

An elevation in the expression of HLA-I antigens in the islet cells of patients with recent-onset type 1 diabetes was first reported approximately 30 years ago [15, 17]. However, neither the significance of this response with respect to disease aetiology nor the mechanism by which it is achieved has been revealed. Moreover, the concept of islet cell HLA-I hyperexpression in type 1 diabetes has recently been challenged, largely on the basis that immunostaining protocols are subject to artefact [18]. In the current study, we counter this by providing firm evidence that the phenomenon occurs in each of the three cohorts of patients we examined, using multiple different HLA-ABC antisera and testing both FFPE and frozen sections. In considering the differing conclusions reached by ourselves and Skog et al [18], we note that the respective immunohistochemistry data are similar, but that Skog et al did not find an elevation at the RNA level in isolated islets from type 1 diabetes patients vs controls. They acknowledged, however, that this is not definitive, since downregulation of HLA-I might occur as a consequence of islet isolation. The studies also differ in that Skog et al [18] studied RNA expression in laser-captured islets from only two patients with type 1 diabetes, whereas we examined islets from many more patients. Therefore, we conclude that hyperexpression of HLA-I is a characteristic feature of islets in human type 1 diabetes, usually linked to remaining insulin, and that it is not an artefact unique to any particular geographical region, mode of tissue preservation or mechanism of pancreas retrieval. These conclusions are supported by evidence of concurrent induction of β2M production with HLA-I, and were confirmed at both the protein level (for all three cohorts) and in RNA studies for the nPOD and DiViD collections, where frozen material was available. The increase at the RNA level was most marked in the DiViD patients, who were all studied soon after disease onset.

We also found that the increased transcription of HLA-I isoforms was not restricted solely to the commonly studied *HLA-ABC* isoforms, but that another, atypical, isoform (*HLA-F*) was also enhanced. This was especially pronounced in the DiViD cohort and was confirmed at the protein level. Of interest, HLA-F expression was localised to the cell surface rather than intracellularly [32]. It has been demonstrated that HLA-F can interact with open-conformation HLA-I heavy chains (without bound peptide), facilitating migration of the complex to the cell surface [33, 34].

Extending these novel data, we also discovered that islet cell hyperexpression of HLA-I can persist, at the protein level, beyond the initial phases of the disease, since it was also seen in patients who had been diagnosed with type 1 diabetes for 11 years prior to death (although it was lost beyond this time). Thus, while there was a tendency towards normalised HLA-I expression over time in the disease course, residual beta cells with elevated HLA-I expression could still be found in some patients long after disease onset. It follows, therefore, that the surviving beta cells in these patients had apparently evaded elimination despite displaying an altered HLA-I phenotype; possibly over many years. The reasons for this are unclear and will require further study. It is also possible that the elevated expression of HLA-I occurs at different times in the disease course and that the islets we examined were at various stages of this progression. However, earlier studies do not support this, since the proportion of ICIs displaying HLA-I hyperexpression in children with recent-onset disease has been reported to be extremely high, suggesting that essentially all such islets hyperexpress HLA-I at diagnosis [15]. We also conclude that beta cells are necessary to initiate and sustain the response, since HLA-I hyperexpression was not present in islets devoid of beta cells in the plane of the section. This may reflect the egress of immune cells from IDIs [11], but it is also possible that elaboration of a diffusible molecule, such as one of the IFNs, by beta cells might drive islet HLA-I hyperexpression in surrounding cells [35, 36], since HLA-I hyperexpression does not correlate directly with insulitis.

Elevated expression of HLA-I has been observed in mouse models of type 1 diabetes, where IFN-γ release from infiltrating immune cells has been shown to be the driver [37, 38]. As noted above, however, we emphasise that, contrary to the situation in mice, hyperexpression of HLA-I can occur in human islets without evidence of insulitis [15, 22, 39, 40]. Of course, this does not exclude the likelihood that, in inflamed islets, the response might be enhanced or sustained by cytokines produced by immune cells.

An important additional new finding in the present work is the striking correlation between the levels of STAT1 measured in the beta cells of patients with type 1 diabetes and HLA-I hyperexpression. This was confirmed in multiple individual islets across a range of different patients and was demonstrated at the protein and RNA levels. STAT1 is a critical protein involved in mediating antiviral responses to IFNs, and its early upregulation in the progression of type 1 diabetes would be expected to place beta cells in a heightened state of responsiveness to these cytokines [41–43].

Although STAT1 was elevated in beta cells soon after disease diagnosis, its expression declined with disease duration, thereby correlating with a similar decline in HLA-I, as discussed above. On this basis, it seems possible that the two may be coordinately regulated or that increased production of HLA-I in beta cells occurs as a consequence of enhanced STAT1 expression. In support of this (and in accord with others [18]), we found no significant increase in levels of the putative HLA-I transcriptional regulator NLRC5 in islets hyperexpressing HLA-I. Thus, an alternative transcriptional regulator must exist in beta cells and this could be STAT1. Importantly, however, cytosolic STAT1 expression was not increased in the non-beta islet endocrine cells in type 1 diabetes, despite these having elevated HLA-I. This implies that a separate mechanism may control HLA expression in these cells, although it is also possible that a modest level of STAT1 activation might occur in the absence of a dramatically altered cytosolic protein level and, in support of this, we did detect nuclear STAT1 in some non-beta cells (Fig. 6a).

Taken together, our observations provide solid evidence that islet cell HLA-I hyperexpression is a genuine pathological feature in type 1 diabetes, and this raises important questions about the role of this phenomenon in disease progression in humans. One hypothesis, which is consistent with our data, proposes that enhanced expression of HLA-I antigens is critical for early disease progression, promoting the effective engagement of influent CD8^+^ cytotoxic T cells specific to defined islet antigens. Finally, our findings also emphasise the complicity of beta cells in their own demise in type 1 diabetes.

## Electronic supplementary material

Below is the link to the electronic supplementary material.

ESM (PDF 1462 kb)

## Abbreviations list

DiViD: Diabetes Virus Detection study
FFPE: Formalin-fixed, paraffin-embedded
HLA-I: HLA class I
ICI: Insulin-containing islet
IDI: Insulin-deficient islet
MFI: Mean fluorescence intensity
NLRC5: NLR family CARD domain containing 5
nPOD: Network for Pancreatic Organ donors with Diabetes
OCT: Optimal cutting temperature
STAT1: Signal transducer and activator of transcription 1
β2M: β2-Microglobulin
