# Supplementary material for: Islet cell hyperexpression of HLA class I antigens: a defining feature in type 1 diabetes
Source: Diabetologia. 2016 Aug 9;59(11):2448–58. doi: 10.1007/s00125-016-4067-4 (PMC5042874; doi:10.1007/s00125-016-4067-4)

ESM Table 1: nPOD Patient Details And Block Information For Those Used In Affymetrix Array And IHC

Case ID	Group	Age (years)	Duration (years)	Block/ Region for FFPE IHC/ IF	Block/ Region for Frozen IF	Block/ Region for utilised for RNA extraction
**6012**	No diabetes control	68		PT 04	PT 01, PT 04, PH 04	PT 02
**6013**	No diabetes control	65		PT 02	PT 02, PH02	PT 04
**6019**	No diabetes control	42		PT 04	PT 04	PT 04
**6024**	No diabetes control	21		PT 08	PT 04	PT 01
**6075**	No diabetes control	16		PB 01	PB 01, PT 04	PB 06
**6099**	No diabetes control	14.2		PB 01	PB 01, PH 04	PB 02
**6102**	No diabetes control	45.1		PT 10		PT 04
**6140**	No diabetes control	38		PB 04	PB 03	PT 04
**6038**	Type 1 diabetes	37.2	20	PB 01, PB 06	PH04, PH 05	PH 06
**6046**	Type 1 diabetes	18.8	8	PB 01, PB 08	Many regions PH, PB, PT	PT 03
**6052**	Type 1 diabetes	12	1	PB 01	PB 04, PB 05, PB 06	PB 03
**6069**	Type 1 diabetes	22.9	7	PB 01	PH 02, PT 04	PT 06
**6070**	Type 1 diabetes	22.6	7	PB 01, PB 02, PT 02	PB 04, PT 04	PT 06
**6084**	Type 1 diabetes	12	4	PB 01	PB 02	PT 02
**6088**	Type 1 diabetes	31.2	5	PB 08, PH 03	PB 04, PT 04	PH 02
**6113**	Type 1 diabetes	13.1	1.58	PB 04; PB 01	PB 04, PT 04	PH 01
**6180**	Type 1 diabetes	27.1	11	PT 02	PT 02	PT 04
**6195**	Type 1 diabetes	19.2	5	PT 02, PT 04	PT01, PH 01	PT 06
**6209**	Type 1 diabetes	5	0.25	PB 04	PB 04, PH 03	PT 01
**6211**	Type 1 diabetes	24	4	PT 04	PT 04, PH 02	PT 04
**6228**	Type 1 diabetes	13	0	PB 04	PB 04, PT 02, PH 02	PT 04
**6243**	Type 1 diabetes	13	5	PT 02	PT 02	PT 02

Key: PB – PanBody; PT – PanTail; PH – PanHead;

Exact regions	Nearby regions	Different regions

ESM Table 2: nPOD, UK and DiViD Patients Additional To Those Cited In Supplementary Table 1.

Case ID	Group	Source	Donor Status	Age (years)	Duration
**6048-04 PT**	No diabetes control	nPOD Biobank (Affymetrix Array)	Organ Donor	30	
**6162-01 PT**	No diabetes control	nPOD Biobank (Affymetrix Array)	Organ Donor	22.7	
**6229-02 PT**	No diabetes control	nPOD Biobank (Affymetrix Array)	Organ Donor	31	
**6251-02 PT**	No diabetes control	nPOD Biobank (Affymetrix Array)	Organ Donor	33	
**6179-04B PB**	No diabetes control	nPOD Biobank (Affymetrix Array)	Organ Donor	21.8	
**333/66**	No diabetes control	UK Pancreas Biobank	Autopsy	16	
**21/89**	No diabetes control	UK Pancreas Biobank	Autopsy	4	
**184/90**	No diabetes control	UK Pancreas Biobank	Autopsy	5	
**330/71**	No diabetes control	UK Pancreas Biobank	Autopsy	47	
**E560**	Type 1 diabetes	UK Type 1 diabetes Biobank	Organ Donor	42	1.5y
**Sc115**	Type 1 diabetes	UK Type 1 diabetes Biobank	Autopsy	1	0 ‘Recent’
**E124B**	Type 1 diabetes	UK Type 1 diabetes Biobank	Autopsy	17	0 ‘Recent’
**DiViD1***	Type 1 diabetes	Norwegian DiViD biobank	Live Donor	25	4 weeks
**DiViD2***	Type 1 diabetes	Norwegian DiViD biobank	Live Donor	24	3 weeks
**DiViD3***	Type 1 diabetes	Norwegian DiViD biobank	Live Donor	34	9 weeks
**DiViD4***	Type 1 diabetes	Norwegian DiViD biobank	Live Donor	31	5 weeks
**DiViD5***	Type 1 diabetes	Norwegian DiViD biobank	Live Donor	24	5 weeks
**DiViD6***	Type 1 diabetes	Norwegian DiViD biobank	Live Donor	35	5 weeks

* Detailed information in Krogvold et al (7).

ESM Table 3: HLA-ABC Antibodies Table

Antibody Details	Species and Clone	Application	Epitope
**Dako HLA-ABC**	Mouse monoclonal; W6/32	IF<br>Not suitable for FFPE sections	A monomorphic epitope on the 45 kDa polypeptide products of the HLA-A, -B and -C loci
**Abcam HLA class I ABC**	Mouse monoclonal; EMR8-5	FFPE<br>WB	Recombinant HLA-A*2402 extracellular domain
**HLA Class I**	Rabbit polyclonal; J.Neefjes, Amsterdam	FFPE	Prepared against a mixture of purified heavy chains of HLA-B7 and HLA-B40. Neefjes JJ, et al (1986) Immunogenetics 23:164-171

ESM Table 4: Antibody Details and Immunocytochemistry Conditions

Primary Antibody	Manufacturer and clone	Antigen Retrieval	Antibody Dilution	Incubation time with primary antibody	Secondary Detection System
**HLA-ABC**	Abcam C#ab70328<br>Mouse monoclonal<br>EMR8-5	10mM citrate pH6.0	1/1500	1h at RT	Dako REAL™ Envision™ Detection System<br>Or Immunofluoroscence staining using anti-mouse IgG (H+L) Alexa Fluor™-conjugated secondary antibodies (1/400 for 1hr)
**HLA Class I**	Neefjes JJ, et al (1986)<br>Rabbit polyclonal	10mM citrate pH6.0	1/500	1hr at RT	Immunofluoroscence staining using anti-rabbit IgG (H+L) Alexa Fluor™ -conjugated secondary antibodies (1/400 for 1hr)
**Insulin**	Dako<br>C#A0564<br>Guinea-pig polyclonal	10mM citrate pH6.0	1/600	1h at RT	Dako REAL™ Envision™ Detection System<br>Or Immunofluoroscence staining using anti-guinea-pig IgG (H+L) Alexa Fluor™ -conjugated secondary antibodies (1/400 for 1hr)
**Glucagon**	Abcam<br>C#ab82270<br>Mouse monoclonal	10mM citrate pH6.0	1/2000	1h at RT	Vector AP-ABC kit combined with Vector Red Substrate kit<br>Or Immunofluoroscence staining using anti-mouse IgG (H+L) Alexa Fluor™-conjugated secondary antibodies (1/400 for 1hr)
**HLA-F**	Abcam<br>C#ab126624<br>Rabbit monoclonal	10mM citrate pH6.0	1/400	1h at RT	Immunofluoroscence staining using anti-rabbit IgG (H+L) Alexa Fluor™ -conjugated secondary antibodies (1/400 for 1hr)
**STAT1**	Abcam C#ab2415<br>Rabbit polyclonal	10mM citrate pH6.0	1/100	1h at RT	Immunofluoroscence staining using anti-rabbit IgG (H+L) Alexa Fluor™ -conjugated secondary antibodies (1/400 for 1hr)

**NLRC5**	Abcam<br>C#ab117624<br>Rabbit polyclonal	10mM citrate pH6.0	1/750	1h at RT	Immunofluoroscence staining using anti-rabbit IgG (H+L) Alexa Fluor™ -conjugated secondary antibodies (1/400 for 1hr)
**B2M**	Cell Signalling<br>C# 12851S<br>Rabbit monoclonal	10mM citrate pH6.0	1/5000	1h at RT	Immunofluoroscence staining using anti-rabbit IgG (H+L) Alexa Fluor™ -conjugated secondary antibodies (1/400 for 1hr)

Supplementary Table 5: Spearman R Values for the STAT1/ HLA correlation analyses

Case	**Total No. ICI Analysed**	**Total No. IDI Analysed**	**Spearmans R**	**P-value**
E560	7	7	0.90330	<0.0001
DIVID 1	7	7	0.82857	0.0003
DIVID 2	7	7	0.89890	<0.0001
DIVID3	7	0	0.7381	
DIVID 4	7	7	0.8257	0.0003
DIVID 5	7	7	0.87692	<0.0001
DIVID 6	8	6	0.79341	0.0007

ESM Fig 1: Isotype control antibody staining using HRP (A) and IF (B) compared with HLA-ABC [EMR8/5] in T1D FFPE tissue and appropriate control tissue. Immunocytochemical staining was performed using either isotype control antibody (mouse IgG1) or HLA-ABC [EMR8-5] under the same experimental conditions. Positive control staining for HLA-ABC is observed in the pancreatic lymph node (PLN). No staining was observed in the isotype control stained PLN or T1D tissue.

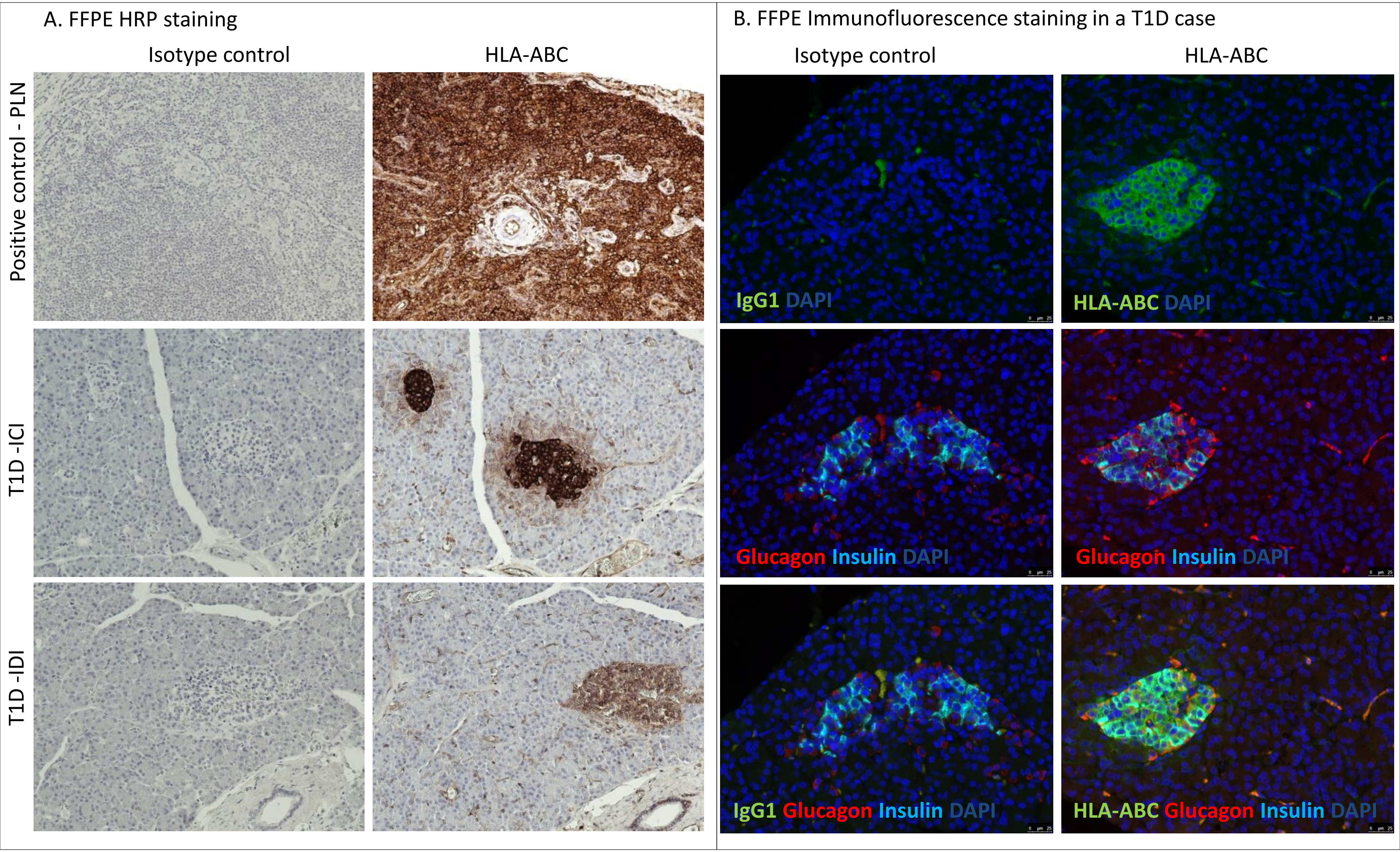

ESM Figure 2: Representative photomicrographs demonstrating normal, elevated and hyper expression of HLA-ABC in pancreas sections from patients with type 1 diabetes. Normal (left) elevated (centre) and hyperexpression (right) of HLA-ABC in islets from the indicated nPOD cases was evaluated in FFPE (A) and frozen (B) sections, using different antibodies in two separate laboratories (Exeter (A) and La Jolla (B)). (C) Concordance between the blinded scoring of islet HLA-ABC expression in the Exeter and La Jolla laboratories for pancreas sections from 8 non-diabetes (ND) controls (upper rows) and 14 type 1 diabetes cases (lower rows).

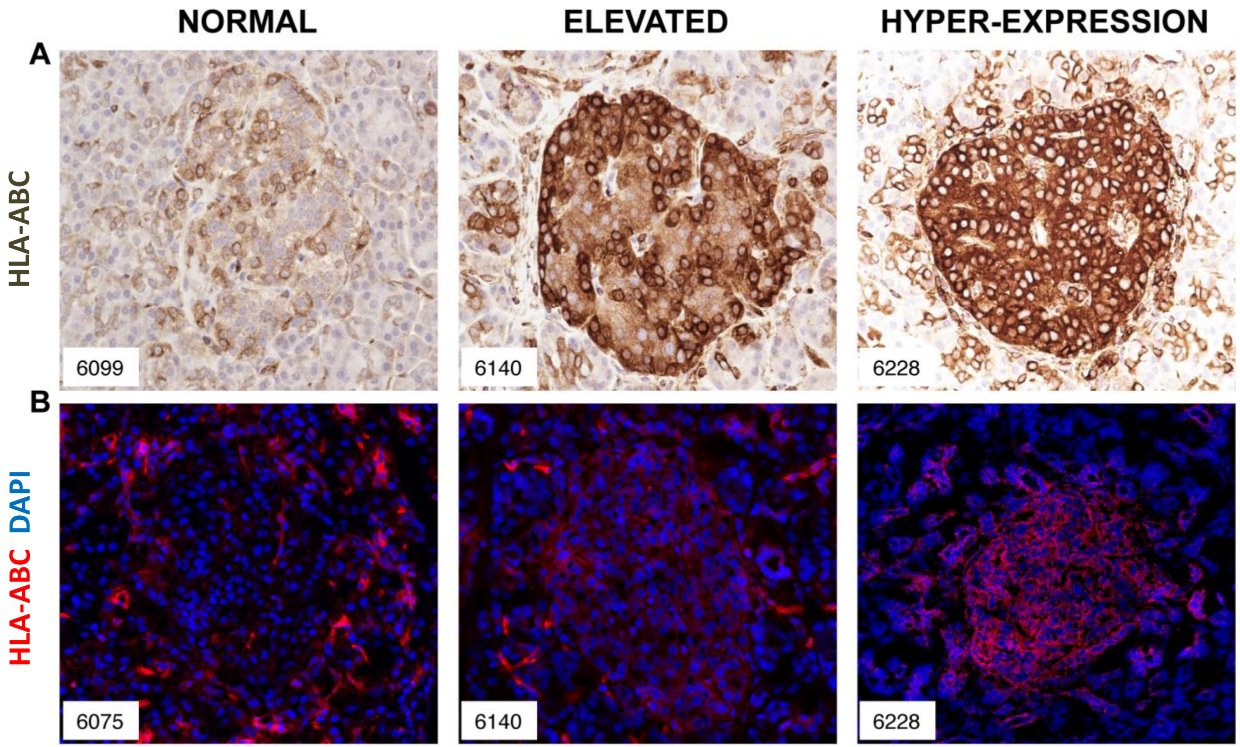

C

ND Ctrls	FFPE (Exeter) Abcam HLA-A, B, C	Frozen (La Jolla) Dako HLA-A, B, C
6013	Normal	Normal
6024	Normal	Normal
6075	Normal	Normal
6012	Normal	Normal
6099	Normal	Normal
6140	Elevated	Elevated
6102	Normal	Normal
6019	Elevated	Elevated

nPOD T1D	FFPE (Exeter) Abcam HLA-A, B, C	Frozen (La Jolla) Dako HLA-A, B, C
6070	Hyper expression	Hyper expression
6084	Hyper expression	Hyper expression
6088	Hyper expression	Hyper expression
6180	Hyper expression	Hyper expression
6243	Hyper expression	Hyper expression
6228	Hyper expression	Hyper expression
6209	Hyper expression	Hyper expression
6038	Elevated	Elevated
6046	Hyper expression	Hyper expression
6069	Normal	Normal
6195	Hyper expression	Hyper expression
6052	Hyper expression	Hyper expression
6211	Hyper expression	Hyper expression
6113	Hyper expression	Hyper expression

ESM Figure 3; Serial sections of the same islet stained with two different HLA Class I antibodies from different species (Rabbit polyclonal – upper panel; Abcam mouse monoclonal (EMR8-5) (lower panel) in FFPE tissue.

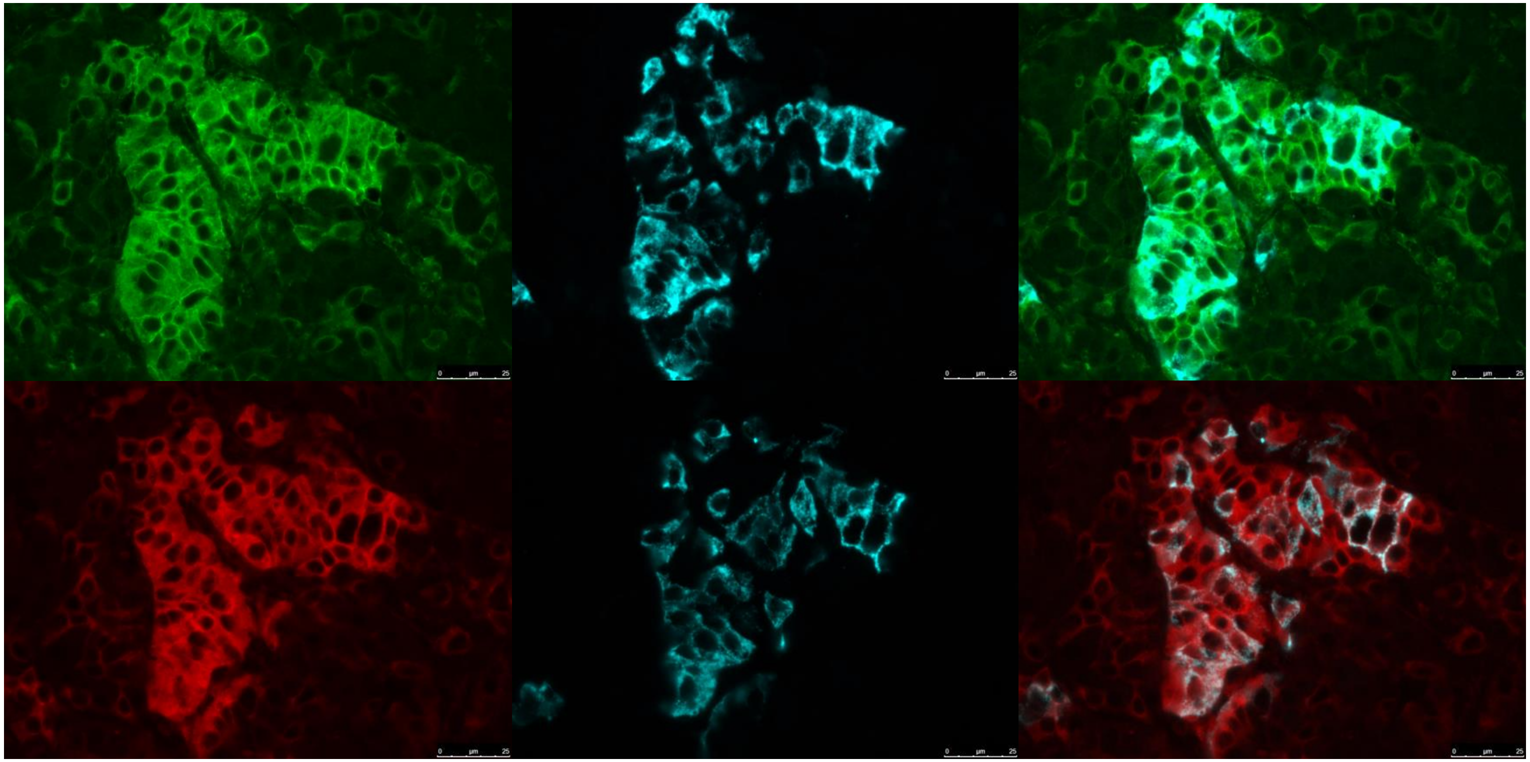

ESM Figure 4: Proportion of ICIs hyperexpressing HLA-ABC (black bars) in pancreas sections from nPOD cases plotted with respect to disease duration of disease (white bars). The Spearman's rank correlation between declining HLA hyperexpression and disease duration was 0.883 (P < 0.0001).

Case ID	Region & Block	Age at death (y)	Duration of disease (y)
**6228**	PB 04	13	0
**6209**	PB 04	5	0.25
**6052**	PB 01	12	1
**6113**	PB 04	13.1	1
**6198**	PT 02	22	3
**6084**	PB 01	12	4
**6211**	PT 04	24	4
**6212**	PB 04	20	5
**6088**	PH 03	31.2	5
**6070**	PT 02	22.6	7
**6046**	PB 08	18.8	8
**6180**	PT 02	27.1	11
**6081**	PB 02	31.5	15
**6038**	PB 06	37.2	20
**6065**	PB 01	79	56

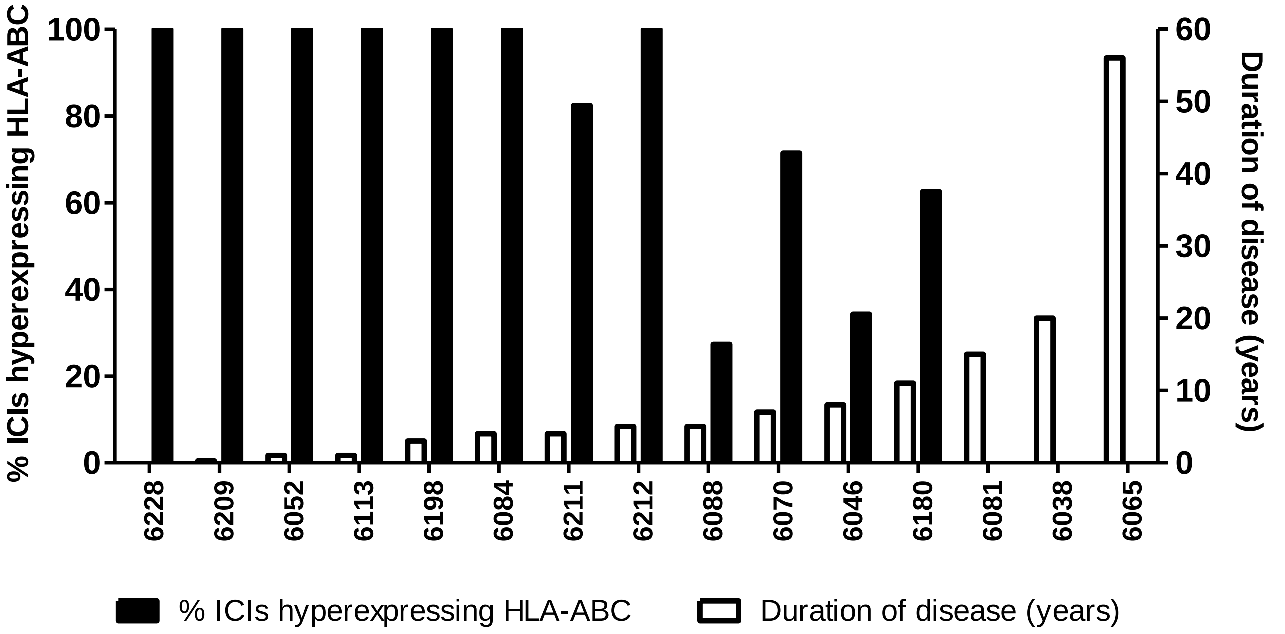

ESM Figure 5: Heat map of relative RNA expression of the *HLA-A, B, C , F, β2M, STAT1* genes examined in nPOD controls and nPOD Type 1 diabetes patients. A comparison with the IHC scoring is shown on the left of the heat map with hyperexpression in black, elevated expression is blue and normal expression is grey. The ICI score relates to the number of residual ICIs in the nearest available block for assessment (+++ >20 ICIs; ++ 10-20 ICIs; + 1-10 ICIs, - No ICIs).

	Case ID	Duration	IHC Score	ICI Score	HLA-A1	HLA-A2	HLA-A3	HLA-A4	HLA-A5	HLA-A6	HLA-A7	HLA-A8	HLA-B1	HLA-B2	HLA-B3	HLA-B4	HLA-B5	HLA-B6	HLA-C1	HLA-C2	HLA-C3	HLA-C4	HLA-C5	HLA-C6	HLA-C7	HLA-F1	HLA-F2	HLA-F3	HLA-F4	HLA-F5	HLA-F6	HLA-F7	B2M	NLRC5	STAT1
Non-diabetic	6013-04PT			+++	55.7	195.3	195.3	130.3	70.7	638.7	67.0	235.4	21.2	170.0	163.0	289.9	147.8	132.2	208.4	204.9	160.9	272.3	95.7	189.3	143.1	72.9	52.5	62.7	62.7	90.2	62.7	8.7	463.4	31.4	122.8
	6024-01PT			+++	58.4	178.5	178.5	97.6	70.7	426.8	99.2	167.6	32.2	223.2	146.0	163.7	146.6	113.8	176.0	217.6	135.8	121.1	45.2	165.6	133.4	48.0	57.5	48.1	48.1	74.5	48.1	8.7	284.5	22.7	182.6
	6075-06PB			+++	76.5	231.3	231.3	195.6	104.5	954.5	91.9	175.4	20.6	142.3	278.1	275.6	288.0	227.8	311.4	371.7	243.4	230.3	94.4	285.0	211.3	79.3	82.2	80.0	80.0	104.8	80.0	11.9	476.3	31.6	255.7
	6012-02PT			+++	50.0	195.2	195.2	208.8	94.3	950.3	98.5	140.2	47.0	205.5	217.3	246.2	246.5	189.5	192.8	219.7	156.5	212.9	79.6	187.1	147.7	67.9	71.2	66.9	66.9	80.9	66.9	9.7	376.6	33.9	249.7
	6099-02PB			+++	69.7	193.8	193.8	181.1	77.7	819.4	74.4	161.7	35.9	165.9	200.8	188.5	232.8	170.3	185.0	199.6	175.1	269.8	87.7	209.6	159.4	59.6	57.0	61.1	61.1	63.2	61.1	9.3	415.3	31.5	227.0
	6102-04PT			+++	87.8	244.4	244.4	188.6	105.9	850.8	96.6	275.8	24.5	181.5	240.9	290.0	235.7	193.0	296.2	371.1	231.2	313.5	73.6	263.3	195.4	61.3	56.1	54.7	54.7	80.1	54.7	7.1	428.6	31.5	193.9
	6140-04PT			+++	214.3	319.1	319.1	222.6	93.3	1123.7	100.2	202.8	38.5	180.1	275.5	313.9	331.1	243.4	227.7	284.6	239.5	357.7	131.5	292.6	212.4	65.0	70.8	63.3	63.3	83.4	63.3	6.5	553.6	23.8	217.1
	6019-04PT			+++	132.9	281.8	281.8	159.7	76.7	817.8	81.3	162.1	37.6	194.1	150.1	220.1	216.5	124.5	211.7	257.9	144.4	270.4	93.4	166.9	132.3	43.8	50.8	45.0	45.0	83.7	45.0	5.9	347.7	25.7	270.0
T1D	6228-04PT	0		++/+++	106.1	263.5	263.5	275.6	116.8	1144.7	127.7	166.5	45.9	166.9	352.5	223.2	368.6	306.9	274.3	331.0	260.1	320.9	133.2	308.8	213.4	67.7	62.6	69.7	69.7	92.0	69.7	8.6	563.4	22.6	249.9
	6209-01PT	0.25		+	41.6	209.9	209.9	178.0	85.4	865.9	65.3	129.4	77.7	240.2	325.1	309.0	368.3	241.6	271.9	329.8	214.0	290.7	68.1	240.8	185.0	65.7	65.0	71.6	71.6	103.8	71.6	11.1	412.6	31.3	385.6
	6052-03PB	1		No ICIs	95.7	269.2	269.2	259.5	112.5	1324.7	119.0	172.3	38.8	137.7	314.7	237.1	323.9	266.6	270.2	291.3	285.7	402.1	113.4	318.1	241.3	75.2	85.8	77.1	77.1	101.0	77.1	7.6	609.0	28.7	266.8
	6113-01PH	1		+	93.6	209.9	209.9	200.5	87.6	934.9	80.8	171.0	26.0	298.9	168.5	259.9	198.1	148.3	273.8	430.4	177.7	335.8	62.2	258.2	173.9	42.7	58.3	55.9	55.9	83.4	55.9	7.2	462.3	31.4	87.9
	6211-04PT	4		+++	93.5	334.3	334.3	269.4	124.0	1321.2	112.2	220.6	41.4	201.8	329.3	282.4	376.6	270.5	268.8	291.6	272.4	465.1	129.5	324.4	237.4	54.2	69.6	66.6	66.6	85.2	66.6	13.1	472.7	30.7	199.9
	6084-02PT	4		No ICIs	44.8	102.5	102.5	93.9	48.5	344.9	49.6	87.6	20.3	176.6	132.5	228.6	172.1	116.4	173.8	204.0	113.9	193.9	77.6	144.3	102.2	50.0	56.0	45.4	45.4	64.6	45.4	15.0	210.6	35.3	109.6
	6243-02PT	5		+++	87.2	311.7	311.7	247.0	98.3	1413.3	99.2	216.7	31.9	253.5	278.8	341.6	295.9	229.7	309.6	383.3	194.4	305.9	56.9	237.2	180.5	55.4	59.4	61.8	61.8	84.1	61.8	10.0	581.4	30.5	215.9
	6195-06PT	5		No ICIs	167.0	266.0	266.0	169.8	88.3	711.7	104.6	232.8	26.9	150.9	159.8	297.1	178.6	134.7	259.4	292.2	168.8	257.3	112.4	201.2	150.2	70.4	66.7	66.8	66.8	88.5	66.8	7.5	607.2	27.3	220.2
	6088-02PH	5		No ICIs	76.7	172.5	172.5	193.0	88.5	922.3	94.0	161.6	20.8	189.2	220.7	272.5	249.3	171.7	195.7	218.8	189.8	235.3	85.4	224.0	163.8	66.3	66.7	62.8	62.8	78.7	62.8	7.5	485.7	26.9	224.2
	6070-06PT	7		++	88.1	231.8	231.8	271.5	95.9	1145.6	112.7	203.0	48.3	150.4	193.2	321.3	246.3	159.2	234.3	274.3	202.2	302.5	114.9	246.4	189.7	61.1	63.0	66.5	66.5	102.7	66.5	7.4	347.3	39.7	160.7
	6069-06PT	7		+++	97.8	233.6	233.6	268.6	97.9	1320.8	121.6	223.0	31.9	192.0	217.8	192.9	232.8	183.0	246.7	291.7	169.2	297.5	76.8	203.3	161.4	60.7	60.2	60.6	60.6	79.7	60.6	8.4	523.2	28.8	181.2
	6046-03PT	8		+++	97.5	242.5	242.5	214.4	89.3	1008.6	98.8	174.9	23.3	195.9	238.7	278.6	260.3	198.7	285.3	321.6	185.9	298.2	100.9	222.8	164.7	65.0	64.4	66.3	66.3	89.8	66.3	11.6	458.1	28.3	216.3
	6180-04PT	11		No ICIs	95.8	294.1	294.1	247.7	104.7	1484.5	102.2	246.6	52.6	218.6	279.8	300.1	315.1	235.0	323.3	375.5	226.4	387.8	123.4	294.0	197.5	61.7	67.6	68.4	68.4	104.5	68.4	7.4	723.4	26.3	254.8
	6038-06PH	20		++	119.6	279.0	279.0	214.6	95.2	1060.6	125.6	210.6	34.2	199.4	225.5	219.0	241.3	185.8	213.9	243.3	216.1	400.6	119.1	263.3	204.6	56.7	56.5	61.8	61.8	85.4	61.8	15.9	463.6	41.6	154.8

ESM Fig 6a: UK case HLA-F expression in ICI and IDI. Immunofluorescence staining of HLA-F (green), insulin (light blue), glucagon (red) and DAPI confirms that HLA-F is expressed at high levels only in insulin positive islets of a UK type 1 diabetes patient (upper panel) and not in the insulin-deficient islet in the type 1 diabetes patient (lower panel).

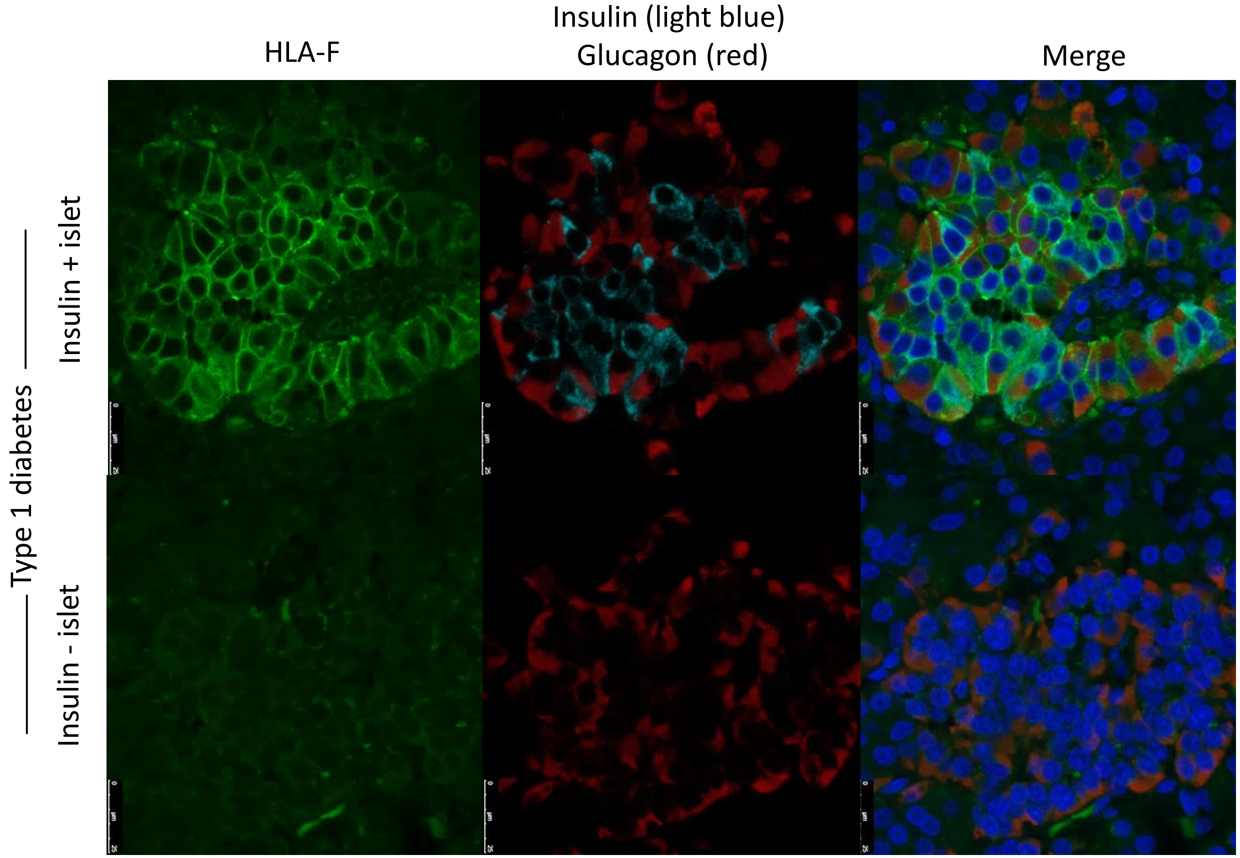

ESM Fig 6b; HLA-ABC and HLA-F co-localise at the plasma membrane, (orange arrow) but HLA-ABC is also expressed in the cytosol in insulin-containing islets in type 1 diabetes (white arrow).

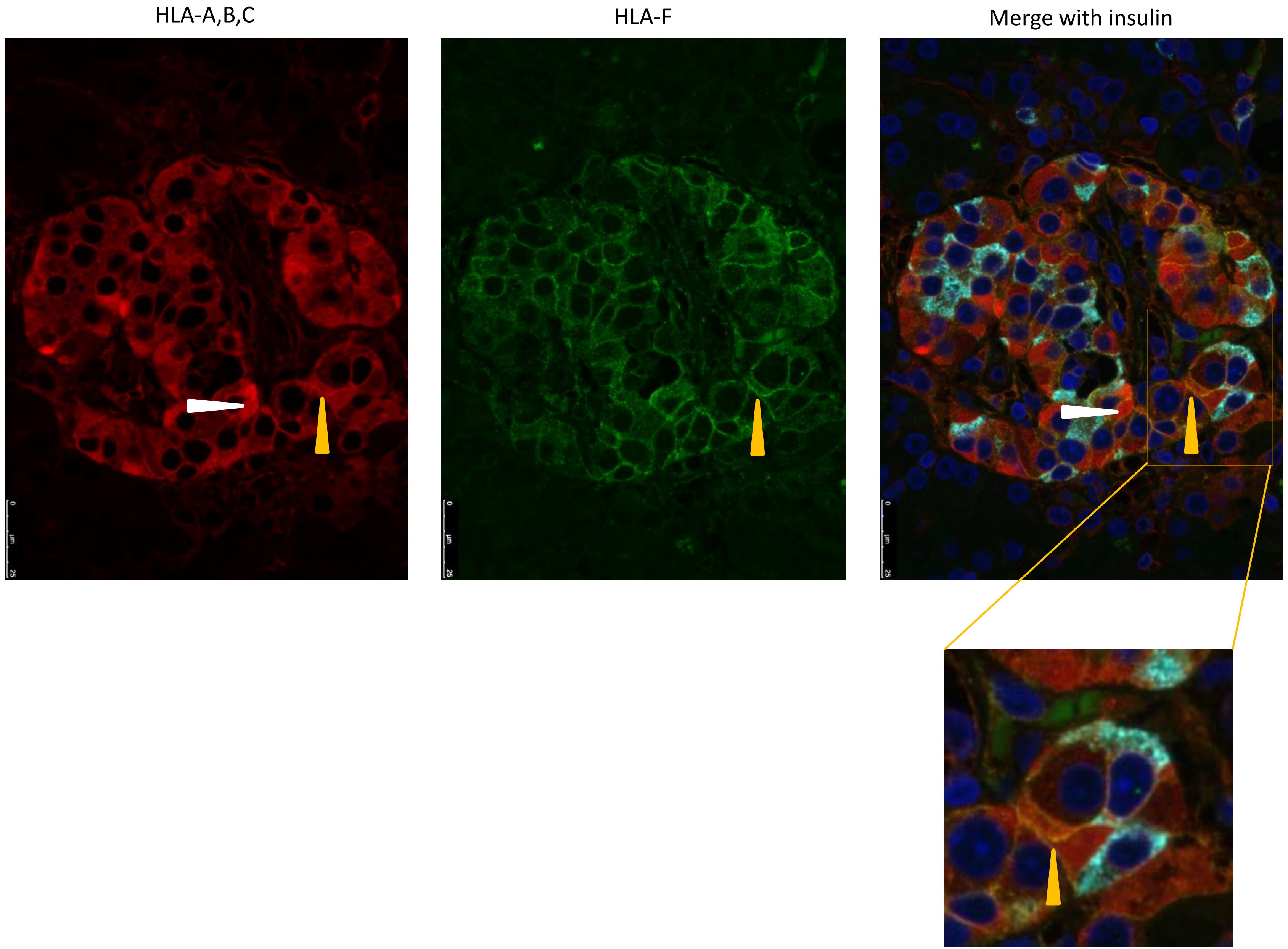

ESM Fig 7 Heat map of relative RNA expression of *STAT1* genes examined in age-matched nPOD controls versus type 1 diabetes DiViD cases.

Case ID		STAT1
Age-matched nPOD cases	6024-01PT	182.6
	6048-04PT	176.8
	6140-04PT	217.1
	6162-01PT	163.3
	6229-02PT	175.3
	6251-02PT	222.6
	6179-04B PB	177.4
DiViD Cases	DiViD-6	737.0
	DiViD-5	889.8
	DiViD-4	550.7
	DiViD-3	1717.6
	DiViD-2	649.6

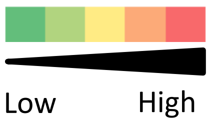

Supplement: Supplementary file 1 — (PDF 1462 kb) [file 125_2016_4067_MOESM1_ESM.pdf]
